# Double Row Hip Abductor Reconstruction with Fasciae Latae Transfer for Severe Trendelenburg after Hip Arthroplasty

**DOI:** 10.3390/jcm13195964

**Published:** 2024-10-07

**Authors:** Bruno Capurro-Soler, Wilson Pizarro-Geraldo, Eduardo Badillo-Pérez, Sebastián González-Vonder Meden, Omar Rivera-Mora, Emerson García-Salas, Francesco Vecchi, Aldo Arguelles

**Affiliations:** 1 Department of Orthopedics and Sports Traumatology, Hospital Ribera IMSKE—European Musculoskeletal Institute, 46024 Valencia, Spain; wilson.epg@gmail.com (W.P.-G.); eduardobadillomd@gmail.com (E.B.-P.); dr.sebastiangvdm@gmail.com (S.G.-V.M.); dott.vecchifrancesco@gmail.com (F.V.); 2 Iberian Group of Hip Preservation Surgery (GIPCA), 4249-004 Porto, Portugal; 3 Muscle and Tendon Study Group (GELMUT) Asociación Española de Artroscopia—AEA, 28003 Madrid, Spain; 4 European Hip Preservation Associates, ESSKA—EHPA, L-1460 Luxembourg, Luxembourg; 5 Department of Orthopaedics and Traumatology, Hospital Civil de Guadalajara, Guadalajara 44200, Mexico; theomarc32@hotmail.com (O.R.-M.); emerson.eegs@gmail.com (E.G.-S.); arguelles@medyarthros.com (A.A.)

**Keywords:** gluteus medius reconstruction, tendinopathy treatment, double-row technique

## Abstract

**Background/Objectives**: Tendinopathy of the gluteus medius and minimus tendons is a primary source of lateral hip pain, ranging from interstitial and partial-thickness tears to complete tears. Treatments include muscle transfers, Achilles tendon allograft procedures, and primary repairs with allografts. This study evaluated the one-year outcomes of gluteus medius and minimus reconstruction using an open double-row technique with a partial tensor fasciae latae transfer for severe Trendelenburg post-total hip arthroplasty. **Methods:** A prospective study involving eight patients who underwent surgery from April to December 2023 was conducted. The surgery involved an open technique with double-row suture reinforcement and tensor fasciae latae autograft. Outcomes were measured using strength, the Harris Hip Score (HHS), 12-Item Short Form Health Survey (SF-12), Hip Outcome Tool (HOT), International Hip Outcome Tool (iHOT), and Visual Analog Scale (VAS). Follow-ups occurred at 1, 3, 6, 9, and 12 months postoperatively. **Results:** At an average follow-up of 7.17 months, significant improvements in both hip function and quality of life were observed. The SF-12 quality of life score increased from 27 preoperatively to 34 by month 12. Hip functionality, as measured by the HHS, showed a marked improvement from 48 to 94 points, particularly after six months. The HOT score for hip functionality rose by 23 points by the third month, reaching an average of 86 points. Similarly, the iHOT score increased from 20 to 83 points starting at month 3, reflecting substantial improvements in hip function. Statistically significant improvements were noted at as early as month 3 (*p* = 0.02), with highly significant gains by month 6 (*p* < 0.01), which remained stable through month 12 (*p* < 0.01). **Conclusions:** Reconstruction of the gluteus medius and minimus tendons using an open double-row technique with a partial tensor fasciae latae transfer significantly enhances hip function and quality of life. Over an average follow-up period of 7.17 months, patients experienced notable improvements. This technique is an effective option for treating lateral hip pain due to tendinopathy.

## 1. Introduction

Tendinopathy and tears of the gluteus medius associated with gluteus minimus pathology is now recognized as a primary source of lateral hip pain [1]. These conditions have increasingly been identified as significant causes of lateral hip pain and dysfunction as well as major contributors to greater trochanteric pain syndrome [2]. Historically, these patients were managed under the assumption of bursitis unresponsive to non-surgical treatment [3].

Histopathological changes observed in the gluteal tendons and bursae of individuals presenting with lateral hip pain are consistent with the degenerative alterations identified in other tendinopathies [4]. These alterations encompass a spectrum ranging from interstitial and partial-thickness tears to complete and retracted tears [5]. The insertions of the gluteus medius and minimus tendons can be effectively assessed using ultrasound (US). Furthermore, magnetic resonance imaging (MRI) proves to be a valuable tool in evaluating both the direct and indirect signs of gluteal tendinopathy in addition to excluding alternative causes of lateral hip pain [6].

Currently, there has been an increase in these tendon repairs; however, outcomes vary according to the severity of muscle atrophy, which has been extensively investigated in the context of rotator cuff injuries [7,8]. Severe fatty degeneration of a muscle does not improve following tendon repair and is correlated with poor functional outcomes [9,10]. The Goutallier classification system has been employed to evaluate fatty degeneration of the gluteal muscles [11]. Based on experiences with shoulder imaging and surgery, it can be anticipated that the degree of pre-therapeutic muscle fatty degeneration will similarly affect gluteal tendon repair outcomes.

The endoscopic repair of gluteus medius tears is well established in the literature, with excellent medium- and long-term outcomes [12]. Patients exhibiting greater fatty infiltration or muscle delamination, particularly in grade 3 and 4 tears (modified Goutallier-Fuchs classification), have reported poorer functional outcomes following endoscopic repair [13]. Consequently, alternative treatments have been proposed, including muscle transfers, Achilles tendon allograft procedures, and primary repairs augmented with allografts [14,15].

Muscle transfers have shown good to excellent results in the early stages; a study by Harper et al. demonstrated improvements in pain and function following the surgical repair of hip abductor tendon injuries with both simple and complex tears. This improvement was observed even during the ongoing healing of the surgical site. MRI findings may remain abnormal for more than a year post-surgery and do not necessarily indicate repair failure [16]. Several case series reporting the use of synthetic tissues, allografts, and autografts for augmentation or reconstruction have shown positive early outcomes. Suppauksorn et al. proposed Superior Gluteal Reconstruction (SGR) utilizing an acellular dermal allograft matrix for the reconstruction of massive and irreparable hip abductor tendon tears, which is the preferred technique of the lead author for large, irreparable gluteus medius tears [17].

A recent study by Burns et al. (2022) introduced a novel approach to abductor reconstruction using partial transfers of the gluteus maximus (Gmax) and tensor fasciae latae (TFL). Specifically, their technique involves transferring the anterior 30% of the Gmax and the posterior 70% of the TFL to the origin of the vastus lateralis. This method aims to simplify the reconstruction process, providing a potentially effective solution for severe abductor deficiencies in revision THA. The technique is performed via a lateral approach, involving minimal dissection of muscle slips and soft tissue, which enhances its practicality [18].

The purpose of this study was to evaluate the one-year outcomes of a case series of patients undergoing reconstruction of the gluteus medius and minimus using the open double-row technique associated with partial transfer of the tensor fasciae latae as a treatment for severe Trendelenburg following total hip arthroplasty or chronic injuries, and the aim was to improve functional recovery.

## 2. Materials and Methods

After we obtained institutional review board approval, clinical data were retrospectively retrieved from a prospectively maintained institutional surgical repository comprising a series of 8 patients who underwent gluteal reconstruction surgery from April 2023 to December 2023. All patients had previously undergone total hip replacement with a modified Hardinge lateral approach and presented with trochanteric pain unresponsive to conservative management, severely limiting daily activities. Although the sample size of 8 patients is relatively small, this study focuses on a highly specialized procedure and a specific patient population with a rare combination of factors, which limits the number of eligible patients. The niche nature of both the condition and the surgical technique, combined with the recruitment timeframe, constrained our ability to include a larger sample. Ultrasonography and 3-Tesla MRI were performed, revealing partial or complete tears of the gluteus medius and minimus. A physical examination showed a Trendelenburg gait, with positive Ossendorf and Lequesne tests for strength and pain. The surgery was performed using an open technique with double-row suture reinforcement augmented with an autograft from a portion of the tensor fasciae latae. Outcomes were evaluated for strength, Harris Hip Score (HHS), Short Form-12 (SF-12), Hip Outcome Tool (HOT), International Hip Outcome Tool (iHOT), and Visual Analog Scale (VAS). Follow-up was conducted at minimum of 3 months and maximum of 12 months, with functional scores being assessed preoperatively and at 1 month, 3 months, 6 months, and 9 months, and 1 year postoperatively.

Statistical analysis was performed using a paired t-test to compare functional scores at each follow-up time point with baseline values, with the significance level set at 0.05.

All patients were evaluated with anteroposterior weight-bearing and Dunn axial radiographs, showing no signs of loosening or malposition of components. Subsequently, ultrasound in the clinical setting revealed extensive bursitis and varying degrees of gluteal tendon injury. A 3-Tesla MRI was then performed and evaluated by a team of musculoskeletal radiology specialists, identifying varying degrees of injury, retraction, and fatty atrophy measured using the Goutallier classification.

### 2.1. Surgical Technique 

**Figure 1:**
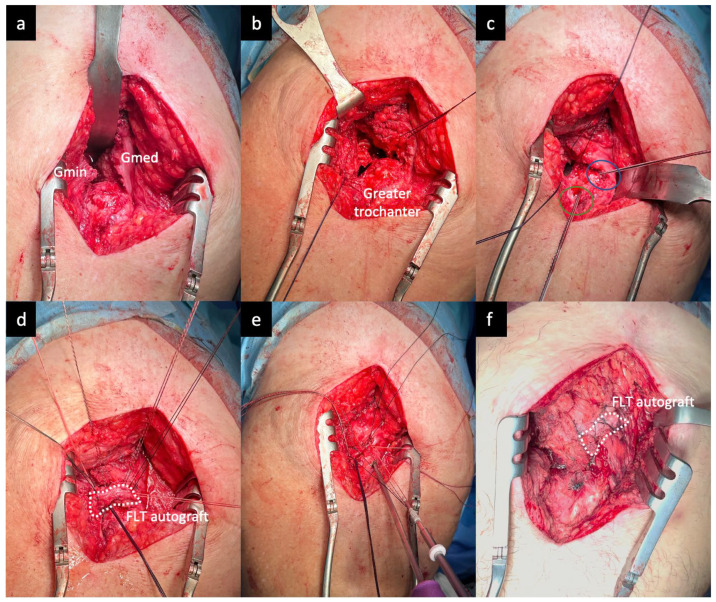
The surgical sequence for gluteal reconstruction: (**a**) The greater trochanter was exposed, and the anatomy of the abductor tendon insertions was delineated. (**b**) The torn gluteus medius and minimus tendons were identified, debrided, and secured with traction sutures, preserving viable tissue. (**c**) Two VERSALOOP™ 2.5 mm (DePuy Synthes and Johnson & Johnson) anchors were placed in the lateral (green circle) and supero-posterior (blue circle) facets near the medial footprint edge. (**d**) A fascia latae autograft (round white dots) was harvested and placed over the defect and footprint. (**e**) One limb of each suture pair (4 pairs) was incorporated into two 4.75 mm PEEK anchors in the lateral row. (**f**) The sutures were sequentially tensioned before inserting the anchors to compress the graft-tendon unit against the footprint. Gmed = gluteus medius, Gmin = gluteus minimus, FLT = fascia latae tendon.

The procedure (Figure 1) was performed using a direct lateral approach with the patient in the lateral decubitus position. An incision was made in the iliotibial band in line with its fibers to allow entry into the peritrochanteric space, followed by a trochanteric bursectomy. The greater trochanter was exposed, and the anatomy of the abductor tendon insertions was delineated. The torn edges of the gluteus medius and minimus tendons were identified and debrided while preserving as much viable tendon as possible. The gluteus minimus was mobilized by releasing the tendon from the underlying capsule and overlying fascia latae. The insertion site on the greater trochanter was then prepared. An anchor configuration was planned for an equivalent double-row transosseous repair based on the tear morphology to recreate the native tendon insertion.

Firstly, two VERSALOOP™ 2.5 mm (DePuy Synthes, Raynham, MA, USA and Johnson & Johnson, New Brunswick, NJ, USA) anchors were inserted into the lateral facet and the superoposterior facet near the medial edge of the footprint. The number of suture anchors depended on the tear size. The sutures and tapes were then passed through the proximal tendon margin in a horizontal mattress configuration using a free needle. An autograft from the fascia latae tendon distal to the injury site was harvested, cut to the required dimensions, and placed over the defect and footprint. With the hip in 20 degrees of abduction and neutral rotation, the medial row sutures and tapes were passed vertically through the graft and tied to compress the patch against the native tendons. One limb of each suture pair (4 pairs) was incorporated into two 4.75 mm PEEK anchors in the lateral row. The sutures were sequentially tensioned before inserting the anchors to compress the graft-tendon unit against the footprint. A similar surgical technique was previously described by Capurro et al., 2023, employing acellular allograft augmentation [19].

Correct tension and positioning were evaluated with passive rotation and abduction maneuvers. Standard irrigation and layered closure were performed.

### 2.2. Surgical Data

All patients underwent open surgery performed by the same surgeon over a period of 1 year. Reconstruction involved augmentation with an autologous graft from the fascia latae, averaging 9.0 cm^2^ in size. In four cases, we used MITEK Stryker anchors of 2.4 mm and 5.0 mm in size. The average blood loss was 125 mL, and the average surgical time was 105 min, with no intraoperative complications.

### 2.3. Postoperative Rehabilitation

Following surgical intervention, patients underwent a rehabilitation process divided into 4 phases. Postoperatively, a hip orthosis was applied, prohibiting active hip abduction and internal rotation, as well as passive hip adduction and external rotation, for 6 weeks. Phase 2 commenced at 6 weeks, progressing patients to full weight-bearing and initiating hip-strengthening exercises as the orthosis was discontinued. Phase 3 began at 12 weeks, allowing ambulation without assistance and gradual return to general activities based on tolerance. At 24 weeks, patients entered phase 4, focusing on strength, endurance, plyometric progression, initiating a running program, and sport-specific exercises. Patients were authorized to discontinue physiotherapy and return to activity or sports between 4 and 6 months depending on their progress.

Patients were scheduled for successive follow-up appointments at 2 weeks, 6 weeks, 3 months, 6 months, 9 months, and 12 months. Demographic data and relevant medical history were recorded in the database. Functional scores were obtained during each evaluation, including VAS, SF-12, HHS, iHOT, and HOS. These data, along with those collected during rehabilitation, were subjected to analysis.

## 3. Results

A total of eight patients underwent open SGR due to severe hip abductor deficiency during the study period and were evaluated at an average of 7.17 months post-initial surgical intervention. The mean age and body mass index were 66.25 years (SD ± 11.11) and 29.4 (SD ± 3.25), respectively, with half of the patients being male (N = 4, 50%). The demographic data of the patients are presented in Table 1, and the physical examination and radiological data are presented in Table 2. All patients exhibited severe Trendelenburg gait at the time of evaluation.

### 3.1. Clinical Outcomes

The clinical outcomes were evaluated using standardized functional, pain, and patient satisfaction scales (Table 3) with a maximum follow-up of 1 year:

- The SF-12 quality of life scale showed an increase from 27 to 34 points.
- The Harris Hip Score (HHS) increased from an average of 48 points preoperatively to 85 points during the subsequent evaluations, with a greater increase being observed after the sixth month.
- Hip Outcome Tool Score (HOT)-specific scales for hip functionality showed an average increase of 23 points, reaching up to 98 points at 9 months of evaluation.
- The International Hip Outcome Tool (iHOT) score increased from an average of 20 points to 90 points starting from the third month.

**Table 3:** Functional scores.

	Preoperative (8P)	Month 3 (8P)	Month 6 (6P)	Month 9 (6P)	Month 12 (2P)
SF12 ^‡^	27	28	30	33	34
HHS ^§^	48	62	90	94	94
HOT ^¶^	23	60	89	98	98
iHOT **	20	65	85	90	95

Functional scale table divided by evaluation month and number of patients. ^‡^ SF12, Short Form-12; ^§^ HHS, Harris Hip Score; ^¶^ HOT, Hip Outcome Tool; ** iHOT, International Hip Outcome Tool.

### 3.2. Statistical Analysis

The paired *t*-test revealed a statistically significant improvement in all functional scores (SF12, HHS, and HOT) at each follow-up time point compared to the baseline (Table 4). This improvement was observed consistently at all follow-up points, indicating that the surgical procedure had a positive impact on functional outcomes.

## 4. Discussion

The most significant finding of our study highlights favorable outcomes at the one-year follow-up, with no recurrences and utilizing a reproducible technique. The objective results include an improved quality of life and enhanced functioning of the intervened hip abductor apparatus.

Furthermore, there was no evidence of new clinical tears during the final follow-up. Currently, evidence supporting surgical intervention with autograft abductor tendon augmentation for primary repairs of the gluteus medius is limited. Patient outcomes at one year postoperatively were comparable to those of patients with partial or full-thickness tears who underwent endoscopic repair with no reported failures [13,20].

In comparison to Burns et al. [18], who reported significant functional gains using gluteus maximus and tensor fascia latae for abductor reconstruction, this study similarly found substantial improvements in hip function and quality of life with the open double-row technique and partial tensor fasciae latae transfer.

It was observed that the tensor fasciae latae was hypertrophied in six (75%) cases, which is similar to the findings reported by Sutter et al. [21], explaining that the tensor fasciae latae often hypertrophies in the context of long-term insufficiency of the gluteus medius and minimus as it compensates in hip abduction.

Poor quality of tissue and tendon retraction poses an increased risk of re-tears. Hence, the use of augmentation is crucial when deciding on abductor apparatus reconstruction. The authors believe that autograft selection provides increased strength, a reduced risk of rejection, and a minimal impact on the donor area without requiring a different approach than the usual one.

Fehm et al. [22] described the use of an Achilles tendon allograft with a calcaneal bone block in seven patients with abductor deficiency following total hip arthroplasty. These patients had an average modified Harris Hip Score (mHHS) of 85.9 at the 2-year follow-up, which is comparable to the average mHHS of 89 in the current study.

Other authors have described repairing the gluteus medius with bioinductive patch augmentation using both open and endoscopic techniques [23]. In patients who underwent abductor tendon repair with augmented suture anchors using synthetic grafts, Ebert et al. [24] reported significant improvements in all scores and mean clinical ratings, including improved hip abductor strength and gait performance in 142 patients.

This study is limited by its small sample size and its design as a case series, which inherently restricts the number of patients. All surgeries were performed by a single surgeon with substantial experience in this technique, which may introduce a level of selection bias. The lack of randomization and the absence of a control group further limit the ability to generalize these findings to a broader population. Additionally, there was no objective assessment of recovery in terms of the median values of hip abductor strength. These factors, combined with the small sample size, mean that the results should be interpreted with caution. Future studies with larger, randomized samples and extended follow-up periods are necessary to more comprehensively evaluate the outcomes and generalizability of this specific technique.

## 5. Conclusions

Reconstruction of the gluteus medius and minimus tendons using an open double-row technique with a partial tensor fasciae latae transfer significantly enhances hip function and quality of life. Over an average follow-up period of 7.17 months, patients experienced notable improvements, with the Harris Hip Score increasing from 48 to 94 points and the iHOT score increasing from 20 to 95 points. This technique is an effective option for treating lateral hip pain due to tendinopathy.

## Figures and Tables

**Table 1:** Demographic data.

Sex	50% (4 males; 4 females)
Age (years)	66.25 ± 11 (range 48–77)
BMI * (kg/m^2^)	29.4 ± 3.25 (range 24.4–35.5)
Laterality (left hip)	5 (62.25%)
Smoking habits	5 (62.25%)
Diabetes	0 (0.0%)
Duration of symptoms before surgery	0 (0%) 1–6 months
	0 (0%) 6–11 months
	5 (62.25%) 1–2 years
	3 (37.75%) >2 years

* BMI, body mass index.

**Table 2:** Preoperative examination.

FADIR (positive)	0%
FABER (positive	0%
OBER (positive)	100%
Trochanteric pain (positive)	100%
Abduction pain (positive)	100%
Trendelenburg	0—Mild
	0—Moderate
	8—Severe
MRI ^†^	
*Affected tendon*	100% (gluteus medius and gluteus minimus)
*Retraction*	No = 1 (12.5%)
	Yes = 7 (87.5%)
*Bursitis*	Mild = 0 (0.0%)
	Moderate = 3 (37.5%)
	Severe = 5 (62.25%)
*Goutallier classification*	1 = 0 (0.0%)
	2 = 4 (50%)
	3 = 4 (50%)
	4 = 0 (0.0%)

^†^ MRI, magnetic resonance imaging.

**Table 4:** Paired *t*-test table for comparison of functional scores.

Time Point	Mean Difference	Standard Error	T-Value	*p*-Value
Month 3	5	1.5	3.33	0.02
Month 6	13	2.0	6.50	<0.01
Month 9	16	2.5	6.40	<0.01
Month 12	17	3.0	5.67	<0.01

## Data Availability

All data supporting the reported results are included within the article and its accompanying tables. No additional datasets were generated or analyzed.

## Abbreviations

Gmax	Gluteus Maximus
Gmed	Gluteus Medius
Gmin	Gluteus Minimus
HOT	Hip Outcome Tool
HHS	Harris Hip Score
iHOT	International Hip Outcome Tool
MRI	Magnetic Resonance Imaging
SD	Standard Deviation
SF-12	12-Item Short Form Health Survey
SGR	Superior Gluteal Reconstruction
TFL	Tensor Fascia Latae
US	Ultrasound
VAS	Visual Analog Scale
